# 4-(Pyrimidin-2-yl)-1-thia-4-aza­spiro­[4.5]decan-3-one

**DOI:** 10.1107/S1600536809049460

**Published:** 2009-11-25

**Authors:** Patrícia D. Neuenfeldt, Bruna B. Drawanz, Wilson Cunico, Edward R. T. Tiekink, James L. Wardell, Solange M. S. V. Wardell

**Affiliations:** a Departamento de Química Orgânica, Universidade Federal de Pelotas (UFPel), Campus Universitário, s/n°, Caixa Postal 354, 96010-900 Pelotas, RS, Brazil; b Fundação Oswaldo Cruz, Instituto de Tecnologia em Fármacos–Farmanguinhos, R. Sizenando Nabuco 100, Manguinhos, 21041-250, Rio de Janeiro, RJ, Brazil; c Department of Chemistry, University of Malaya, 50603 Kuala Lumpur, Malaysia; d Centro de Desenvolvimento Tecnológico em Saúde (CDTS), Fundação Oswaldo Cruz (FIOCRUZ), Casa Amarela, Campus de Manguinhos, Av. Brasil 4365, 21040-900 Rio de Janeiro, RJ, Brazil; e CHEMSOL, 1 Harcourt Road, Aberdeen AB15 5NY, Scotland

## Abstract

The title compound, C_12_H_15_N_3_OS, features an envelope conformation for the 1,3-thia­zolidin-4-one ring with the S atom as the flap atom. The pyrimidine ring is almost orthogonal to the 1,3-thia­zolidin-4-one ring as indicated by the N—C—C—N torsion angle of −111.96 (18)°. Supra­molecular dimers are formed in the crystal structure through the agency of C—H⋯O contacts occurring between centrosymmetrically related mol­ecules. These are linked into supra­molecular tapes along [100] *via* C—H⋯S contacts.

## Related literature

For the biological activity of thia­zolidinones, see: Cunico *et al.* (2008*a* ▶); Solomon *et al.* (2007 ▶); Kavitha *et al.* (2006 ▶); Sharma *et al.* (2006 ▶); Ravichandran *et al.* (2009 ▶); Rao *et al.* (2004 ▶). For background to the synthesis, see: Cunico *et al.* (2008*b* ▶); Rawal *et al.* (2008 ▶). For related studies on the synthesis and biological evaluation of thia­zolidinones, see: Cunico *et al.* (2006 ▶, 2007 ▶).
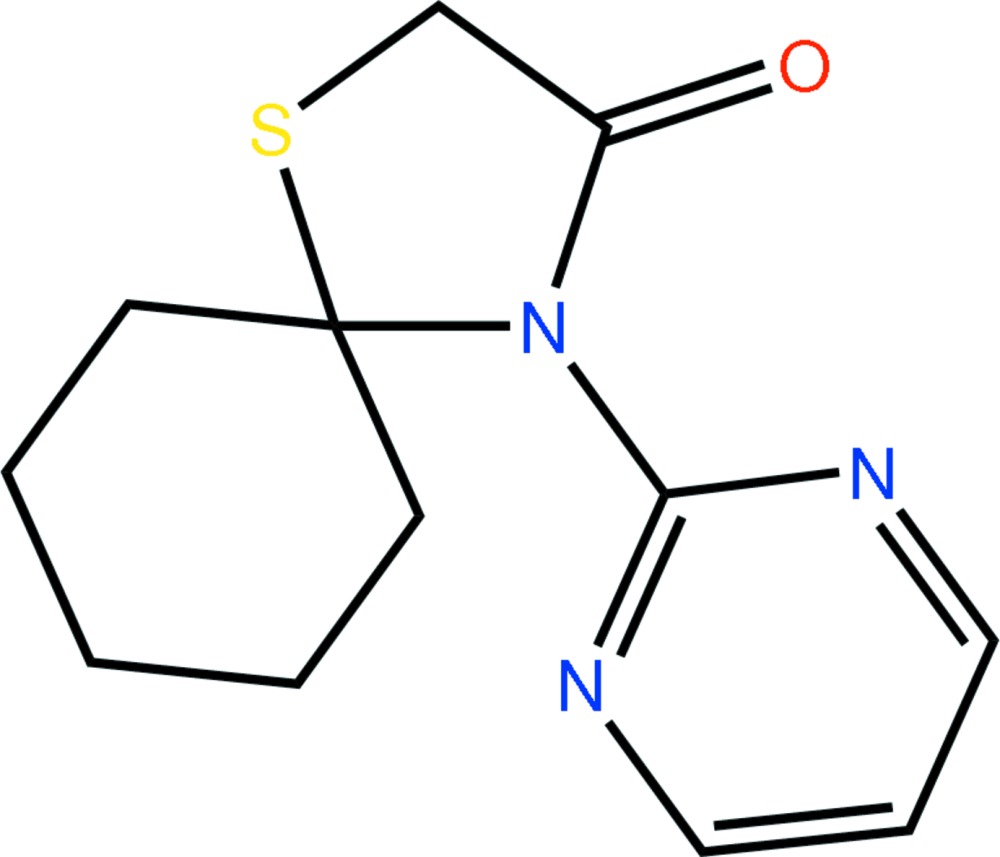


## Experimental

### 

#### Crystal data

C_12_H_15_N_3_OS
*M* *_r_* = 249.33
Monoclinic, 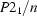

*a* = 6.2466 (2) Å
*b* = 8.6748 (2) Å
*c* = 22.0439 (6) Å
β = 95.698 (1)°
*V* = 1188.61 (6) Å^3^
*Z* = 4
Mo *K*α radiation
μ = 0.26 mm^−1^
*T* = 120 K
0.26 × 0.22 × 0.14 mm

#### Data collection

Nonius KappaCCD area-detector diffractometer
Absorption correction: multi-scan (*SADABS*; Sheldrick, 2003 ▶) *T*_min_ = 0.658, *T*_max_ = 0.746
14004 measured reflections
2661 independent reflections
2227 reflections with *I* > 2σ(*I*)
*R*_int_ = 0.054

#### Refinement

*R*[*F* ^2^ > 2σ(*F* ^2^)] = 0.038
*wR*(*F* ^2^) = 0.113
*S* = 1.14
2661 reflections
155 parameters
H-atom parameters constrained
Δρ_max_ = 0.36 e Å^−3^
Δρ_min_ = −0.40 e Å^−3^

### 

Data collection: *COLLECT* (Hooft, 1998 ▶); cell refinement: *DENZO* (Otwinowski & Minor, 1997 ▶) and *COLLECT*; data reduction: *DENZO* and *COLLECT*; program(s) used to solve structure: *SHELXS97* (Sheldrick, 2008 ▶); program(s) used to refine structure: *SHELXL97* (Sheldrick, 2008 ▶); molecular graphics: *DIAMOND* (Brandenburg, 2006 ▶); software used to prepare material for publication: *publCIF* (Westrip, 2009 ▶).

## Supplementary Material

Crystal structure: contains datablocks global, I. DOI: 10.1107/S1600536809049460/hg2601sup1.cif

Structure factors: contains datablocks I. DOI: 10.1107/S1600536809049460/hg2601Isup2.hkl

Additional supplementary materials:  crystallographic information; 3D view; checkCIF report

## Figures and Tables

**Table 1:** Hydrogen-bond geometry (Å, °)

*D*—H⋯*A*	*D*—H	H⋯*A*	*D*⋯*A*	*D*—H⋯*A*
C10—H10a⋯S1^i^	0.99	2.80	3.4765 (13)	126
C10—H10b⋯O1^ii^	0.99	2.44	3.361 (2)	155

Symmetry codes: (i) 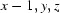; (ii) 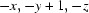.

**Fig. 1.**
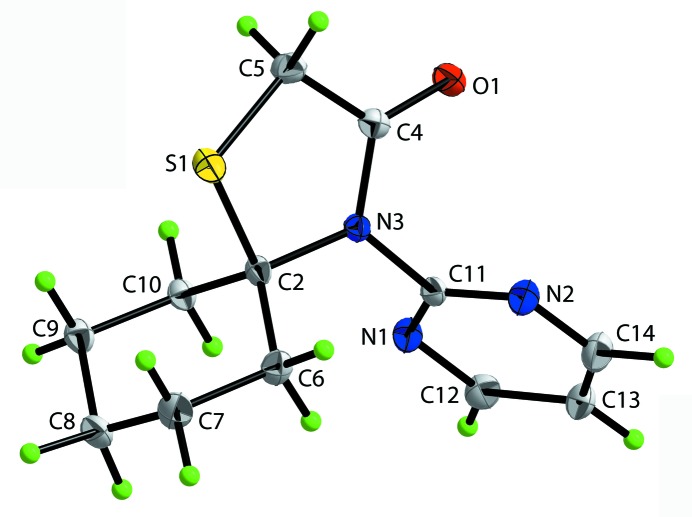
Molecular structure of (I) showing atom-labelling scheme and displacement ellipsoids at the 50% probability level.

**Fig. 2.**
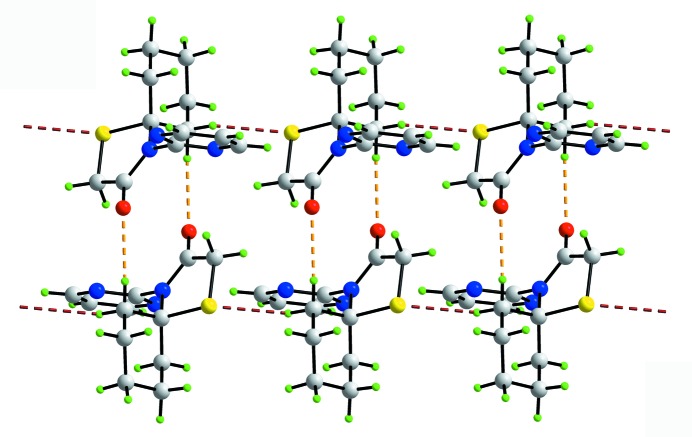
Supramolecular tape formation in (I) whereby dimeric aggregates sustained by C—H···O (orange dashed lines) contacts are linked via C—H···S contacts (brown dashed lines) along [1 0 0].

## supplementary crystallographic information

### Comment

Thiazolidinones constitute an important group of heterocyclic compounds (Cunico *et al.*, 2008*a*), having valuable biological uses, for example, as anti-malarial (Solomon *et al.*, 2007), anti-microbial (Kavitha *et al.*, 2006), anti-inflammatory (Sharma *et al.*, 2006), and anti-viral agents, especially as anti-HIV agents (Ravichandran *et al.*, 2009; Rao *et al.*, 2004). The main synthetic routes to 1,3-thiazolidin-4-ones involve three components (an aldehyde, an amine and mercaptoacetic acid), either in a one- or two-step process (Cunico *et al.*, 2008*a*; Rawal *et al.*, 2006). In continuation of our research on thiazolidinones, (Cunico *et al.*, 2006; Cunico *et al.*, 2007: Cunico *et al.*, 2008*b*), we report the structure of the title compound, 1-thia-4-azaspiro[4.5]decan-3-one, (I).

The molecule structure of (I) shows the five-membered 1,3-thiazolidin-4-one ring to adopt an envelope conformation with the S1 atom as the flap atom. The cyclohexyl ring adopts a regular chair conformation. The pyrimidine is twisted out of the plane of the 1,3-thiazolidin-4-one ring as seen in the value of the C4–N3–C11–N1 torsion angle of -111.96 (18) °. When viewed along the plane through the N1, C2, C4 and C5 atoms, the molecule, with the exception of the S1 atom, has approximate mirror symmetry.

In the crystal structure, centrosymmetric pairs associate *via* C—H···O contacts to form dimers, Table 1. The dimeric aggregates are linked into a supramolecular tape aligned along [1 0 0] *via* C—H···S contacts, Table 1 and Fig.2. The pivotal role of the C10-methylene group is noted in the stabilization of the crystal structure as each of the C10-bound H atoms forms a significant intermolecular contact.

### Experimental

A mixture of 2-aminopyrimidine (1 mmol), cyclohexanone (2 mmol) and mercaptoacetic acid (3 mmol) in toluene (35 ml) was heated at 403 K with a Dean-Stark trap for 16 h. The reaction was cooled, washed with NaHCO_3_ (3 *x* 20 ml), and dried with MgSO_4_. The crude product was washed with a hot solvent mixture of hexane/ethyl acetate (9:1) and recrystallized from EtOH. Yield 80%. m. pt. 475–476 K. ^1^H NMR (200 MHz, CDCl_3_): δ 8.76 (s, 2H, aryl), 7.22 (s, 1H, aryl), 3.66 (s, 2H, H5), 2.21–1.57 (m, 10H, CH_2_) p.p.m. ^13^C NMR (100 MHz, CDCl_3_): δ 172.4 (CO), 158.5, 158.4, 119.3 (aryl), 75.7 (C2), 39.5 (CH_2_), 38.0 (CH_2_), 31.9 (C5), 24.4 (CH_2_), 23.6 (CH_2_) p.p.m.

### Refinement

The C-bound H atoms were geometrically placed with C—H = 0.95–0.99 Å, and refined as riding with *U_iso_*(H) = 1.2*U*_eq_(C).

### Crystal data

**Table:**

C_12_H_15_N_3_OS	*F*(000) = 528
*M**_r_* = 249.33	*D*_x_ = 1.393 Mg m^−^^3^
Monoclinic, *P*2_1_/*n*	Mo *K*α radiation, λ = 0.71073 Å
Hall symbol: -P 2yn	Cell parameters from 7514 reflections
*a* = 6.2466 (2) Å	θ = 2.9–27.5°
*b* = 8.6748 (2) Å	µ = 0.26 mm^−^^1^
*c* = 22.0439 (6) Å	*T* = 120 K
β = 95.698 (1)°	Block, colourless
*V* = 1188.61 (6) Å^3^	0.26 × 0.22 × 0.14 mm
*Z* = 4	

### Data collection

**Table:**

Nonius KappaCCD area-detector diffractometer	2661 independent reflections
Radiation source: Enraf Nonius FR591 rotating anode	2227 reflections with *I* > 2σ(*I*)
10 cm confocal mirrors	*R*_int_ = 0.054
Detector resolution: 9.091 pixels mm^-1^	θ_max_ = 27.5°, θ_min_ = 3.0°
φ and ω scans	*h* = −8→7
Absorption correction: multi-scan (*SADABS*; Sheldrick, 2003)	*k* = −10→11
*T*_min_ = 0.658, *T*_max_ = 0.746	*l* = −28→28
14004 measured reflections	

### Refinement

**Table:**

Refinement on *F*^2^	Secondary atom site location: difference Fourier map
Least-squares matrix: full	Hydrogen site location: inferred from neighbouring sites
*R*[*F*^2^ > 2σ(*F*^2^)] = 0.038	H-atom parameters constrained
*wR*(*F*^2^) = 0.113	*w* = 1/[σ^2^(*F*_o_^2^) + (0.0508*P*)^2^ + 0.5459*P*] where *P* = (*F*_o_^2^ + 2*F*_c_^2^)/3
*S* = 1.14	(Δ/σ)_max_ < 0.001
2661 reflections	Δρ_max_ = 0.36 e Å^−^^3^
155 parameters	Δρ_min_ = −0.40 e Å^−^^3^
0 restraints	Extinction correction: *SHELXL97* (Sheldrick, 2008), Fc^*^=kFc[1+0.001xFc^2^λ^3^/sin(2θ)]^-1/4^
Primary atom site location: structure-invariant direct methods	Extinction coefficient: 0.012 (2)

### Special details

**Table:**

Geometry. All s.u.'s (except the s.u. in the dihedral angle between two l.s. planes) are estimated using the full covariance matrix. The cell s.u.'s are taken into account individually in the estimation of s.u.'s in distances, angles and torsion angles; correlations between s.u.'s in cell parameters are only used when they are defined by crystal symmetry. An approximate (isotropic) treatment of cell s.u.'s is used for estimating s.u.'s involving l.s. planes.
Refinement. Refinement of *F*^2^ against ALL reflections. The weighted *R*-factor *wR* and goodness of fit *S* are based on *F*^2^, conventional *R*-factors *R* are based on *F*, with *F* set to zero for negative *F*^2^. The threshold expression of *F*^2^ > 2σ(*F*^2^) is used only for calculating *R*-factors(gt) *etc*. and is not relevant to the choice of reflections for refinement. *R*-factors based on *F*^2^ are statistically about twice as large as those based on *F*, and *R*- factors based on ALL data will be even larger.

### Fractional atomic coordinates and isotropic or equivalent isotropic displacement parameters (Å^2^)

**Table:**

	*x*	*y*	*z*	*U*_iso_*/*U*_eq_	
S1	0.30913 (6)	0.59301 (5)	0.14394 (2)	0.02184 (17)	
O1	0.19102 (19)	0.33684 (15)	0.00174 (6)	0.0215 (3)	
N1	−0.2660 (2)	0.24603 (17)	0.08348 (7)	0.0189 (3)	
N2	0.0587 (2)	0.09881 (17)	0.08941 (8)	0.0206 (3)	
N3	0.0732 (2)	0.36704 (16)	0.09593 (6)	0.0147 (3)	
C2	0.0699 (2)	0.47007 (19)	0.14937 (7)	0.0143 (3)	
C4	0.1922 (2)	0.40748 (19)	0.04984 (8)	0.0161 (4)	
C5	0.3225 (3)	0.5513 (2)	0.06440 (8)	0.0207 (4)	
H5A	0.2630	0.6384	0.0390	0.025*	
H5B	0.4737	0.5349	0.0561	0.025*	
C6	0.0987 (3)	0.3792 (2)	0.20899 (8)	0.0194 (4)	
H6A	0.2353	0.3207	0.2110	0.023*	
H6B	−0.0204	0.3041	0.2098	0.023*	
C7	0.1015 (3)	0.4855 (2)	0.26444 (8)	0.0239 (4)	
H7A	0.1098	0.4224	0.3020	0.029*	
H7B	0.2311	0.5517	0.2665	0.029*	
C8	−0.0989 (3)	0.5870 (2)	0.26138 (8)	0.0230 (4)	
H8A	−0.2266	0.5217	0.2655	0.028*	
H8B	−0.0852	0.6607	0.2959	0.028*	
C9	−0.1310 (3)	0.6762 (2)	0.20155 (8)	0.0186 (4)	
H9A	−0.0129	0.7518	0.1999	0.022*	
H9B	−0.2683	0.7338	0.1996	0.022*	
C10	−0.1346 (2)	0.5672 (2)	0.14669 (8)	0.0159 (4)	
H10A	−0.2612	0.4983	0.1461	0.019*	
H10B	−0.1486	0.6284	0.1086	0.019*	
C11	−0.0534 (2)	0.22881 (19)	0.08959 (8)	0.0144 (3)	
C12	−0.3796 (3)	0.1143 (2)	0.07570 (9)	0.0214 (4)	
H12	−0.5323	0.1194	0.0722	0.026*	
C13	−0.2817 (3)	−0.0280 (2)	0.07264 (8)	0.0215 (4)	
H13	−0.3630	−0.1200	0.0658	0.026*	
C14	−0.0591 (3)	−0.0296 (2)	0.08007 (9)	0.0246 (4)	
H14	0.0131	−0.1256	0.0785	0.030*	

### Atomic displacement parameters (Å^2^)

**Table:**

	*U*^11^	*U*^22^	*U*^33^	*U*^12^	*U*^13^	*U*^23^
S1	0.0154 (2)	0.0251 (3)	0.0259 (3)	−0.00679 (16)	0.00633 (17)	−0.00990 (19)
O1	0.0249 (6)	0.0233 (7)	0.0168 (6)	−0.0011 (5)	0.0045 (5)	−0.0020 (5)
N1	0.0163 (7)	0.0159 (8)	0.0243 (8)	−0.0001 (5)	0.0017 (6)	−0.0026 (6)
N2	0.0177 (7)	0.0163 (8)	0.0279 (9)	−0.0001 (5)	0.0023 (6)	−0.0012 (6)
N3	0.0144 (6)	0.0138 (7)	0.0161 (7)	−0.0015 (5)	0.0033 (5)	−0.0025 (6)
C2	0.0123 (7)	0.0152 (8)	0.0154 (8)	−0.0015 (6)	0.0015 (6)	−0.0035 (7)
C4	0.0137 (7)	0.0186 (9)	0.0161 (9)	0.0030 (6)	0.0016 (6)	0.0023 (7)
C5	0.0195 (8)	0.0224 (9)	0.0204 (9)	−0.0039 (7)	0.0037 (7)	0.0002 (8)
C6	0.0230 (8)	0.0171 (9)	0.0173 (9)	0.0021 (7)	−0.0019 (7)	0.0006 (7)
C7	0.0331 (10)	0.0221 (10)	0.0156 (9)	0.0008 (8)	−0.0021 (7)	0.0002 (8)
C8	0.0297 (9)	0.0236 (10)	0.0168 (9)	−0.0026 (7)	0.0071 (7)	−0.0045 (8)
C9	0.0156 (7)	0.0195 (9)	0.0208 (9)	0.0015 (6)	0.0028 (6)	−0.0026 (7)
C10	0.0133 (7)	0.0171 (9)	0.0173 (9)	0.0016 (6)	0.0017 (6)	−0.0012 (7)
C11	0.0158 (7)	0.0144 (8)	0.0130 (8)	−0.0010 (6)	0.0015 (6)	−0.0017 (6)
C12	0.0166 (8)	0.0230 (10)	0.0243 (10)	−0.0034 (7)	0.0008 (7)	−0.0023 (8)
C13	0.0243 (9)	0.0166 (9)	0.0235 (10)	−0.0050 (7)	0.0017 (7)	−0.0041 (7)
C14	0.0243 (9)	0.0164 (9)	0.0334 (11)	0.0015 (7)	0.0036 (8)	−0.0018 (8)

### Geometric parameters (Å, °)

**Table:**

S1—C5	1.8004 (19)	C6—H6B	0.9900
S1—C2	1.8494 (16)	C7—C8	1.527 (3)
O1—C4	1.224 (2)	C7—H7A	0.9900
N1—C11	1.330 (2)	C7—H7B	0.9900
N1—C12	1.347 (2)	C8—C9	1.525 (3)
N2—C11	1.328 (2)	C8—H8A	0.9900
N2—C14	1.339 (2)	C8—H8B	0.9900
N3—C4	1.363 (2)	C9—C10	1.533 (2)
N3—C11	1.436 (2)	C9—H9A	0.9900
N3—C2	1.480 (2)	C9—H9B	0.9900
C2—C10	1.527 (2)	C10—H10A	0.9900
C2—C6	1.528 (2)	C10—H10B	0.9900
C4—C5	1.507 (2)	C12—C13	1.382 (3)
C5—H5A	0.9900	C12—H12	0.9500
C5—H5B	0.9900	C13—C14	1.384 (2)
C6—C7	1.530 (3)	C13—H13	0.9500
C6—H6A	0.9900	C14—H14	0.9500
			
C5—S1—C2	93.63 (8)	H7A—C7—H7B	108.0
C11—N1—C12	115.20 (15)	C9—C8—C7	111.57 (14)
C11—N2—C14	115.15 (15)	C9—C8—H8A	109.3
C4—N3—C11	118.58 (14)	C7—C8—H8A	109.3
C4—N3—C2	119.29 (14)	C9—C8—H8B	109.3
C11—N3—C2	122.06 (13)	C7—C8—H8B	109.3
N3—C2—C10	112.34 (13)	H8A—C8—H8B	108.0
N3—C2—C6	111.29 (14)	C8—C9—C10	111.08 (15)
C10—C2—C6	110.16 (13)	C8—C9—H9A	109.4
N3—C2—S1	102.80 (10)	C10—C9—H9A	109.4
C10—C2—S1	110.96 (12)	C8—C9—H9B	109.4
C6—C2—S1	109.06 (11)	C10—C9—H9B	109.4
O1—C4—N3	124.04 (15)	H9A—C9—H9B	108.0
O1—C4—C5	123.77 (15)	C2—C10—C9	111.29 (13)
N3—C4—C5	112.18 (15)	C2—C10—H10A	109.4
C4—C5—S1	107.29 (12)	C9—C10—H10A	109.4
C4—C5—H5A	110.3	C2—C10—H10B	109.4
S1—C5—H5A	110.3	C9—C10—H10B	109.4
C4—C5—H5B	110.3	H10A—C10—H10B	108.0
S1—C5—H5B	110.3	N2—C11—N1	128.08 (15)
H5A—C5—H5B	108.5	N2—C11—N3	115.10 (13)
C2—C6—C7	111.52 (15)	N1—C11—N3	116.80 (14)
C2—C6—H6A	109.3	N1—C12—C13	122.23 (16)
C7—C6—H6A	109.3	N1—C12—H12	118.9
C2—C6—H6B	109.3	C13—C12—H12	118.9
C7—C6—H6B	109.3	C12—C13—C14	116.59 (16)
H6A—C6—H6B	108.0	C12—C13—H13	121.7
C8—C7—C6	111.59 (15)	C14—C13—H13	121.7
C8—C7—H7A	109.3	N2—C14—C13	122.69 (17)
C6—C7—H7A	109.3	N2—C14—H14	118.7
C8—C7—H7B	109.3	C13—C14—H14	118.7
C6—C7—H7B	109.3		
			
C4—N3—C2—C10	101.41 (16)	C2—C6—C7—C8	54.95 (19)
C11—N3—C2—C10	−75.57 (19)	C6—C7—C8—C9	−53.8 (2)
C4—N3—C2—C6	−134.54 (15)	C7—C8—C9—C10	54.38 (19)
C11—N3—C2—C6	48.48 (19)	N3—C2—C10—C9	−178.32 (14)
C4—N3—C2—S1	−17.93 (17)	C6—C2—C10—C9	57.00 (18)
C11—N3—C2—S1	165.09 (12)	S1—C2—C10—C9	−63.87 (16)
C5—S1—C2—N3	19.77 (12)	C8—C9—C10—C2	−56.35 (18)
C5—S1—C2—C10	−100.52 (12)	C14—N2—C11—N1	2.2 (3)
C5—S1—C2—C6	137.96 (12)	C14—N2—C11—N3	−176.42 (16)
C11—N3—C4—O1	3.4 (2)	C12—N1—C11—N2	−0.6 (3)
C2—N3—C4—O1	−173.65 (15)	C12—N1—C11—N3	177.99 (15)
C11—N3—C4—C5	−177.71 (14)	C4—N3—C11—N2	66.8 (2)
C2—N3—C4—C5	5.2 (2)	C2—N3—C11—N2	−116.21 (17)
O1—C4—C5—S1	−170.17 (14)	C4—N3—C11—N1	−111.96 (18)
N3—C4—C5—S1	10.98 (18)	C2—N3—C11—N1	65.0 (2)
C2—S1—C5—C4	−18.17 (13)	C11—N1—C12—C13	−1.7 (3)
N3—C2—C6—C7	178.46 (13)	N1—C12—C13—C14	2.1 (3)
C10—C2—C6—C7	−56.27 (18)	C11—N2—C14—C13	−1.6 (3)
S1—C2—C6—C7	65.74 (15)	C12—C13—C14—N2	−0.4 (3)

### Hydrogen-bond geometry (Å, °)

**Table:**

*D*—H···*A*	*D*—H	H···*A*	*D*···*A*	*D*—H···*A*
C10—H10a···S1^i^	0.99	2.80	3.4765 (13)	126
C10—H10b···O1^ii^	0.99	2.44	3.361 (2)	155

Symmetry codes: (i) *x*−1, *y*, *z*; (ii) −*x*, −*y*+1, −*z*.
